# Implementation insights into bridging the gaps in isoniazid preventive therapy uptake in adults with HIV

**DOI:** 10.5588/ijtldopen.25.0802

**Published:** 2026-09-15

**Authors:** N. Nevrekar, A. PM, N. Gupte, G. Dhumal, P. Kulkarni, R. Shrisunder, K. Ubale, S. Munde, A. Gupta, N. Suryavanshi

**Affiliations:** 1 Johns Hopkins Center for Infectious Diseases in India, Pune, Maharashtra, India;; 2 B.J. Government Medical College-Johns Hopkins University Clinical Research Site, Pune, India;; 3 Byramjee Jeejeebhoy Government Medical College and Sassoon General Hospital, Pune, India;; 4 Division of Infectious Diseases, Johns Hopkins University School of Medicine, Baltimore, MD, USA.

**Keywords:** tuberculosis, India, TPT, HIV care, consolidated framework for implementation research, CFIR

## Abstract

**BACKGROUND:**

TB remains the leading opportunistic infection among people living with HIV (PLHIV). Isoniazid preventive therapy (IPT) is a key strategy for TB prevention, yet uptake remains suboptimal. This study examined barriers and facilitators to IPT uptake among adult PLHIV and health care providers (HCPs) using the Consolidated Framework for Implementation Research (CFIR).

**METHODS:**

A cross-sectional, mixed-methods study was conducted at the BJ Government Medical College ART Centre, Pune, India, from May 2022 to July 2023. Qualitative data from 11 in-depth interviews (5 PLHIV, 6 HCPs) were thematically analysed using MAXQDA-2022, and quantitative data from 201 PLHIV were analysed using R.

**RESULTS:**

Among 201 PLHIV, 50% (n = 100) completed IPT, 38% (n = 77) missed doses, and 23% (n = 47) reported side effects. Integrated analysis identified drug stock-outs as the dominant system-level barrier, alongside facility-level constraints such as limited counselling due to staff shortages. Individual-level barriers included fear of side effects and time constraints. Facilitators included effective counselling, family support, perceived preventive benefits, motivation, and trust in providers. HCPs emphasised assertive counselling, routine IPT prescription, and regular follow-up as critical to improving IPT completion.

**CONCLUSION:**

Ensuring an uninterrupted drug supply, sufficient staffing, and structured counselling is essential for strengthening IPT implementation within HIV programmes.

The dual burden of TB and HIV infection remains a major global public health challenge.^1–3^ Among people living with HIV (PLHIV), TB is the most common opportunistic infection and the leading cause of death.^4–6^ PLHIV are estimated to have an 18-fold higher risk of developing active TB compared with those without HIV.^7^ In 2023, 10.8 million people developed TB globally, including 0.662 million (6.1%) PLHIV, resulting in 1.25 million TB-related deaths, of which 12.9% occurred among PLHIV.^8^ India accounts for 26% of the global TB burden and has over 2.4 million PLHIV, ranking third worldwide in HIV burden.^8,9^ The WHO recommends isoniazid preventive therapy (IPT) as a key TB preventive therapy.^10^ When given with antiretroviral therapy (ART), IPT substantially lowers TB incidence among PLHIV. Despite strong evidence of effectiveness, IPT uptake in India and other low- and middle-income countries remains suboptimal.^11,12^ Reported barriers include fear of isoniazid (INH) resistance, side effects, inadequate knowledge, drug stock-outs, and poor adherence.^13,14^

Given the limited empirical research in India, it is crucial to identify both barriers and facilitators that influence IPT implementation in programmatic settings. This study aimed to explore the determinants of IPT implementation among PLHIV and HCPs using a mixed-methods approach. The findings provide evidence to inform policies and targeted interventions to improve IPT uptake and adherence. As India adopts WHO-recommended newer and shorter TB preventive regimens, these insights can support their effective implementation in settings with a high HIV–TB burden.^15,16^

## METHODS

A cross-sectional mixed-methods study was conducted at the ART centre, BJ Government Medical College-Sassoon General Hospital (BJGMC-SGH), Pune, one of the largest public-sector HIV treatment facilities in western India, from May 2022 to July 2023.

### Study population and procedures

We included adult PLHIV (≥18 years) initiated on IPT since January 2019, and health care providers (HCPs), including medical officers, nurses, pharmacists, and counsellors. PLHIV diagnosed with active TB before or during IPT were excluded. The study was conducted sequentially, comprising an initial qualitative phase followed by a quantitative phase. The qualitative component explored and contextualised key implementation barriers to IPT uptake. In-depth interviews (IDIs) were conducted with HCPs and PLHIV using purposive sampling by a trained behavioural scientist in a private room at the ART centre using an interview guide. The interview guide covered TB knowledge, its prevention, factors influencing IPT initiation and adherence, and perceptions of IPT delivery. Interviews were audio-recorded, transcribed verbatim, inductively coded, and thematically analysed. IDIs were continued until no new themes emerged, with thematic saturation determined through iterative team review of transcripts; identified themes were consistent with the existing literature.^17^

Findings from the qualitative phase informed the development of the semi-structured interview schedule (SSIS), supplemented by a review of relevant literature. Content validity was established through expert review by clinicians and implementation researchers experienced in HIV–TB care, who assessed the relevance, clarity, and contextual appropriateness. The SSIS was piloted and refined based on feedback before finalisation. In the quantitative phase, adult PLHIV were recruited using systematic random sampling, with every fifth consecutive patient presenting for ART refill at the BJGMC ART Centre invited to participate. The quantitative component enabled estimation of IPT uptake and quantification of the barriers identified in the qualitative phase, providing population-level insight into implementation challenges.

### Outcome definitions

In this study, the IPT uptake was defined as initiation of IPT among eligible adult PLHIV, classified further into completion or non-completion of the regimen. IPT initiation was referred to the receipt of at least one dose of IPT as documented in ART records. IPT completion was defined as documented completion of the 6-month IPT regimen, while non-completion was referred to as initiation without completion of the prescribed IPT regimen, as per the ART records.

### Data analysis

Qualitative data from IDIs were analysed thematically using MAXQDA-2022 (VERBI Software). Themes were reviewed and interpreted through investigator consensus. Quantitative data were summarised using descriptive statistics in R (version 4.2.0). We compared the socio-demographic variables for IPT uptake between completers and non-completers using the Wilcoxon rank sum test, Pearson’s χ² test, and Fisher’s test. Participants receiving IPT at the time of assessment were excluded from analysis. The Consolidated Framework for Implementation Research (CFIR) was used to examine multilevel factors influencing IPT uptake among PLHIV across five domains: Intervention Characteristics, Outer Setting, Inner Setting, Characteristics of Individuals, and Process. Qualitative and quantitative findings were integrated during interpretation and organised according to relevant CFIR domains, ensuring coherence and triangulation.

### Ethical statement

Ethics approval was obtained from the Institutional Review Board of the BJGMC clinical research site and the Johns Hopkins University (USA) IRB. Written informed consent was obtained from all participants after explaining the study procedures, ensuring confidentiality, and clarifying that their participation was voluntary and they could withdraw at any time.

## RESULTS

In the qualitative component of the study, 11 IDIs were conducted (5 PLHIV, 6 HCPs). The PLHIV participants comprised three men and two women, with a median age of 42 years. Their education ranged from middle school to higher secondary level. One participant completed IPT, two had ongoing IPT, one failed to complete it, and one did not initiate it. Among HCPs (median age 40 years), three were male (counsellor, pharmacist, data manager) and three female (medical officer, two nurses), with work experience ranging from a few months to 23 years.

A total of 201 PLHIV were analysed in the quantitative component of the study; the median age was 44 years (interquartile range: 33–49). More than half of the participants were female (56%, n = 112), 53% (n = 106) were married, and 87% (n = 175) were literate. Most participants (73%, n = 147) belonged to the upper-lower socio-economic class, 78% (n = 157) were employed, and 59% (n = 118) reported substance use (Supplementary Data Table S1). These variables did not differ significantly between participants who completed IPT and those who did not.

### CFIR domains

1. Intervention characteristics (subconstructs: Relative advantage, Complexity, Evidence Strength and Quality)

Quantitatively, awareness of TB and IPT was high among PLHIV, with 97% reporting knowledge of TB, 94% awareness of IPT (Supplementary Data Table S1), and 96% recognising the preventive benefits of IPT (Table 2). However, only 79% correctly identified the 6-month treatment duration (Table 1). PLHIV and HCPs both expressed concerns about side effects, INH resistance, and treatment complexity, which contextualised the quantitative findings. These findings indicate that high awareness and recognition of benefits did not translate into IPT initiation or continuation, as safety concerns undermined treatment persistence (Table 2).

2. Outer setting (subconstructs: Patient needs and resources, External policies and incentives)

**Table 1.** Facilitators of isoniazid preventive therapy (IPT) uptake.

Facilitators of IPT uptake	n (%)
Knowledge about TB (N = 201)	
Do you know what TB is?	199 (99%)
Is TB communicable?	177 (88%)
Is TB curable?	190 (95%)
Is TB preventable?	194 (97%)
PLHIV have more chances of getting TB	186 (93%)
Knowledge about IPT (N = 201)	
Heard about TB prevention medicines	198 (99%)
Possible benefits of IPT are known (reduces TB risk)	193 (96%)
IPT course duration is known (6 months)	159 (79%)
Other facilitators (N = 201)	
Patient-provider engagement/counselling from HCPs	178 (89%)
Self-motivation	177 (88%)
Family support	152 (76%)
Nutritional assistance	34 (17%)

PLHIV = people living with HIV; HCPs = health care providers.

**Table 2.** Participant quotes on barriers and facilitators of IPT uptake across CFIR domains.

CFIR domain	Theme	Participant quote	Participant type
Intervention characteristics	Side effects and fear of IPT	When I started my medicines (IPT), I started feeling dizzy, so I stopped taking it.	PLHIV, 42 years
		Newly diagnosed patients don’t understand what exactly is going on... They become non-compliant even after a lot of counselling. Psychiatric help is required as they have depression and anxiety.	Medical officer, 26 years
	Knowledge about TB and IPT	TB infection can be spread from one person to another through sputum or air.	PLHIV, 35 years
Outer setting	Medicine stock-outs	There was a shortage of isoniazid for 4–5 months. Patients can usually buy other medicines from outside, but isoniazid is not available in pharmacies, so it’s difficult for them to continue treatment.	Pharmacist, 48 years
		……then the next month there was stock out, they (HCPs) advised to buy from outside (IPT)	PLHIV, 24 years
Inner setting	Training of health care providers (HCPs)	No, I haven’t received any specific training. I joined and started directly doing this.	Medical officer, 26 years
	Staff shortage	As there are too many patients in the queue, we cannot speak to them too much; a maximum of 4–5 minutes are given for one IPT patient.	Health care provider, 40 years
	Counselling	If I was explained the side effects, how and at what time to consume tablets, then I would not have discontinued the medicines.	PLHIV, 42 years
Characteristics of individuals	Time constraints	My son is handicapped, so I must do both household and outside work. I finish dinner late, around 11 pm, and sometimes skip my medicine because it gets too late.	PLHIV, 40 years
	Motivation and support	The doctor told me not to skip the medicine, because it would prevent TB. In the future, it would be beneficial, so I took it.	PLHIV, 42 years
Process	Patient–provider engagement and trust in HCPs	If proper counselling is done and benefits are explained, patients don’t find it difficult to take the medicines.	Pharmacist, 48 years
		IPT is given when ART is started; it’s a good thing if we continue it regularly.	Counsellor, 45 years
	Definitive prescription and assertive counselling	When I came here, they directly told me about my report, explained everything, completed the paperwork, and started the treatment immediately.	PLHIV, 35 years
		At the start, patients think they have TB because they’re given medicines, so we explain that it’s preventive – to ensure they don’t get TB in the future.	Staff nurse, 40 years

CFIR = Consolidated Framework for Implementation Research; IPT = isoniazid preventive therapy; ART = antiretroviral therapy; PLHIV = people living with HIV.

External contextual factors substantially influenced IPT uptake. Drug stock-outs were the most frequently reported reason for IPT non-completion, cited by 82% of participants who did not complete IPT. Verification with ART records confirmed treatment interruption during periods of IPT unavailability. Travel distance and transportation challenges accounted for 8% (Table 3). During IDIs, HCPs reported recurrent IPT supply interruptions that disrupted treatment continuity, while PLHIV described missed doses due to medication unavailability (Table 2). Converging evidence from both data sources identified drug stock-outs as the dominant barrier to IPT completion, indicating weaknesses in system-level supply management rather than patient unwillingness.

3. Inner setting (subconstructs: Structural characteristics, Implementation climate, Available resources, Access to knowledge and information)

**Table 3.** Barriers to isoniazid preventive therapy (IPT) uptake.

Barriers to IPT uptake	
Reasons for not completing IPT^A^ (N = 101)	n (%)
Stock-outs of IPT tablets	83 (82%)
Medicines interrupted by health care providers (due to adverse drug reaction or TB diagnosis while on IPT)	10 (9%)
Medicines stopped on their own (due to adverse drug reaction or perceived side effects of IPT)	8 (8%)
Long distance from the ART centre, transportation issue	8 (8%)
Insufficient counselling regarding IPT (regarding the duration, side effects and its management, and benefits)	7 (7%)
Lack of support or motivation to take medicines	6 (6%)
Pill burden	6 (6%)
Limited understanding of the importance of completing the IPT	5 (6%)
Other (medical conditions, fear of ADR, forgetfulness, etc.)	10 (10%)

ART = antiretroviral therapy; ADR = adverse drug reactions.

A Multiple responses were permitted; values represent n (%) of non-completers who cited each reason.

The challenges within the ART centre influenced IPT delivery, with 7% of PLHIV reporting inadequate counselling and 6% citing lack of motivation or support to continue IPT (Table 3). Although these proportions were modest, qualitative findings provided critical context. HCPs described a lack of periodic refresher training, high patient load, staff shortages, lengthy TB screening procedures, and limited time for counselling as persistent challenges that constrained their ability to provide individualised education. PLHIV echoed these concerns, noting limited counselling about the IPT side effects. These findings suggest that gaps in counselling and support were driven by structural and resource constraints within ART centres, rather than a lack of provider engagement (Table 2).

4. Characteristics of individuals (subconstructs: Knowledge and beliefs about intervention, Self-efficacy, Individual stage of change

At the individual level, IPT side effects (23%), pill burden (6%), and factors such as medical conditions, fear of adverse drug reactions, and forgetfulness (10%) emerged as reasons for treatment interruption and non-completion of IPT (Supplementary Data Table S1; Table 3). Self-motivation (88%) and family support (76%) emerged as strong facilitators (Table 1). Qualitative narratives expanded on these findings, with PLHIV expressing self-motivation to protect themselves and their families from TB, and describing cultural and spiritual practices such as prayer, yoga, and dietary changes as sources of adherence support. Together, these findings show that readiness for preventive therapy was present and could be strengthened through patient-centred strategies that address practical barriers and reinforce motivation.

5. Process (subconstructs: Engaging, Executing, Reflecting and Evaluating)

Patient–provider engagement was widely recognised as essential for IPT uptake (89%) – see Table 1. The participants unanimously reported that counselling before IPT initiation improved their understanding of the regimen and motivated them to adhere. Trust in HCPs emerged as a key facilitator of adherence, which encouraged them to initiate and complete IPT. Providers emphasised that assertive prescribing and consistent counselling supported sustained IPT adherence. These findings underscore the importance of patient–provider interaction and effective implementation for IPT provision (Table 2).

## DISCUSSION

The CFIR-guided mixed-methods study provided an integrated understanding of multilevel factors influencing IPT uptake among PLHIV in Pune, India. Despite the inclusion of IPT in India’s national HIV programme since 2017, IPT initiation and completion remain suboptimal nationally (approximately 43%–45%), highlighting persistent implementation challenges.^7,18,19^

The study findings indicated that both PLHIV and HCPs were aware of IPT benefits, reflecting their perceived preventive value against TB. However, only three-quarters of participants correctly identified the 6-month treatment duration, indicating an important knowledge gap between general awareness and treatment understanding. This gap is programmatically important, as an incomplete understanding of regimen requirements may undermine treatment persistence and completion even among motivated individuals. Similar findings have been reported from India and Uganda, suggesting that information provision alone is insufficient without clear, reinforced messaging throughout the treatment course.^13,20,21^ Fear of adverse drug effects, concerns about INH resistance, and perceived treatment complexity were key deterrents to initiating and completing IPT, consistent with studies from other high-burden countries like India and Africa.^22–24^

The most frequently cited external barrier in our study was INH stock-out, suggesting that discontinuation was primarily driven by system-level constraints rather than patient behaviour. Similar challenges have been reported from other sites in India and sub-Saharan Africa, where weak logistics and poor supply chain management reduced IPT uptake.^13,25–27^ Although forecasting and procurement processes were not directly examined, the convergence of quantitative findings and provider narratives points to vulnerabilities in maintaining uninterrupted IPT supply within the ART programme. This divergence underscores the added value of a mixed-method approach in distinguishing patient-reported experiences from underlying system-level causes. Programmatically, these findings suggest that ensuring uninterrupted IPT availability may have a greater impact on completion than patient-focused interventions alone.

Our study reported that high patient volume, limited staffing, and insufficient counselling time were key constraints to effective IPT implementation at the facility level. HCPs reported difficulty balancing clinical responsibilities with preventive counselling, a finding consistent with other Indian ART settings.^20,25^ Similar system-level challenges were documented in East African ART centres, where insufficient staff and competing clinical priorities hindered IPT uptake.^26,28^ HCPs reported limited opportunities for refresher training, a finding consistent with studies from India and Nigeria, which identified periodic training, capacity building, and updated clinical guidelines as crucial to sustaining IPT implementation.^13,29^ Collectively, these findings suggest that improving IPT outcomes requires not only adherence to guidelines but also alignment of preventive services with adequate staffing, streamlined workflows, and effective counselling.

At the individual level, time constraints and pill burden contributed to non-completion, consistent with prior studies.^13,26–30^ Our findings align with other Indian studies, which showed that side effects often lead to treatment interruptions, highlighting the ongoing need for clinical monitoring.^13,30,31^ Encouragingly, intrinsic motivation, trust in HCPs, and family support emerged as strong facilitators. Most participants expressed a desire to protect themselves and their families from TB. Comparable evidence from India and sub-Saharan Africa shows that patient–provider trust and perceived benefits of IPT enhance adherence.^25,26,32^ These findings indicate that readiness for preventive therapy exists and can be leveraged through supportive, patient-centred approaches.

The implementation process, particularly patient counselling and engagement, was central to IPT success. Most participants reported that counselling from HCPs supported treatment continuation, with IDIs indicating that supportive counselling facilitated both IPT initiation and completion. The study findings reinforce that consistent counselling enhances understanding of IPT benefits and addresses treatment interruptions due to side effects. Consistent with findings from Indian and African settings, provider confidence in prescribing and counselling emerged as critical drivers of sustained IPT adherence in our study, highlighting the important role of patient–provider interaction in effective IPT implementation.^20,25,26,28,33,34^

This study was conducted in a routine programmatic ART setting with stakeholder engagement, enhancing its policy and practice relevance. A key strength of this study is the integrated CFIR framework, which systematically identifies the complex, multilevel drivers of IPT uptake among PLHIV, bridging the gap between clinical care and real-world implementation. Cross-verification of participant responses with ART records strengthened data reliability.

Limitations include the single-site design, which limits generalisability, and the small qualitative sample, which may have constrained thematic breadth. The quantitative component had limited power to detect small effect sizes. Although recall bias was possible, verification with clinical records minimised this risk. Findings may not be generalisable to children and adolescents living with HIV, and dedicated studies are warranted in these populations. Despite these limitations, the study provides context-specific evidence to inform the implementation of IPT among PLHIV.

We make the following recommendations. Facility-level monitoring of INH stock status could be established at ART centres, with designated responsibility for timely reporting of low stock levels to prevent treatment interruptions. Where feasible, stock tracking tools integrated into existing ART programme systems could support early identification of supply gaps. In addition, coordination between HIV and TB services at the facility and district levels needs to be further strengthened through review meetings between ART centre staff and TB programme personnel, to jointly review IPT uptake, stock availability, and follow-up processes, thereby reinforcing shared accountability for IPT implementation. Periodic refresher training for HCPs could be conducted to reinforce IPT eligibility criteria, side effect management, and effective counselling practices. In addition, strengthening follow-up and patient support after IPT initiation, such as structured counselling at subsequent visits, may help sustain treatment continuation. Collectively, these recommendations highlight practical, system-focused strategies that are feasible within routine programme settings and directly aligned with the barriers identified in this study.

## CONCLUSION

The findings reveal that awareness and understanding of IPT were high. Several system and individual-level barriers, such as INH stock-outs and inadequate counselling due to staffing shortages and time constraints, limited IPT uptake. However, motivational counselling, family support, and trust in HCPs emerged as key facilitators of adherence.

## Supplementary Material
