# Supplementary material for: Implementation insights into bridging the gaps in isoniazid preventive therapy uptake in adults with HIV
Source: IJTLD Open. 2026 Sep 15;3(9):545–51. doi: 10.5588/ijtldopen.25.0802 (PMC13577205; doi:10.5588/ijtldopen.25.0802)
Supplement: Supplementary file 1 [file ijtldopen25-0802_supplementarydata1.pdf]

Table S1: Sociodemographic and Clinical Characteristics

Characteristics	Total (N=201)	IPT Uptake[$]		p-value
		Not completed (N=101)	Completed (N=100)	
**Age (IQR)**	44 (33-49)	42 (34-49)	44 (32-49)	>0.9
**Gender:** Female	112 (56%)	54 (53%)	58 (58%)	0.5
**Education:** Literate	175 (87%)	87 (86%)	88 (88%)	0.8
**Employment Status:** Employed	157 (78%)	82 (81%)	75 (75%)	0.3
**Marital Status**				
Single	24 (12%)	12 (12%)	12 (12%)	0.2
Married	106 (53%)	60 (59%)	46 (46%)	
Widow/er	58 (29%)	24 (24%)	34 (34%)	
Divorce/separated	13 (6.5%)	5 (5.0%)	8 (8.0%)	
**Socioeconomic Status- Modified Kuppuswamy Scale (MKS)***				
Lower (<5)	11 (5.5%)	8 (7.9%)	3 (3.0%)	0.4
Upper lower (5-10)	147 (73%)	72 (71%)	75 (75%)	
Lower middle (11-15)	27 (13%)	12 (12%)	15 (15%)	
Upper middle (16-25)	15 (7.5%)	8 (7.9%)	7 (7.0%)	
Upper (26-29)	1 (0.5%)	1 (1.0%)	0 (0%)	
**Substance Use****				
Yes	118 (59%)	62 (61%)	56 (56%)	0.4
**Reported Side Effects of IPT[@]**				
Yes	47 (23%)	20 (20%)	27 (27%)	0.2
**Missed IPT medication**				
Yes	77 (38%)	49 (49%)	28 (28%)	0.003
**Family history of TB**				
Yes	54 (27%)	23 (23%)	31 (31%)	0.2
**Knowledge about TB[+]**				
Yes	195 (97%)	98 (97%)	97 (97%)	>0.9
**Knowledge about TB Prevention[§]**				
Yes	189 (94%)	95 (94%)	94 (94%)	>0.9
**ART Duration (years)**				
<=5 years	101 (50%)	52 (51%)	49 (49%)	0.8
**Past History of TB**				
Yes	81 (40%)	40 (40%)	41 (41%)	0.9

[$]IPT Uptake: Please refer to the Methods section for definitions.
***MKS: Modified Kuppuswamy Scale- an urban socioeconomic status scale based on education, occupation, and income. (*Sood P et al. Int J Community Med Public Health. 2022 Oct;9(10):3841-3844* http://www.ijcmph.com)
****Substance use: self-reported current use of alcohol and tobacco (within last 30 days).
[@]IPT side effects: self-reported using a semi-structured questionnaire listing common adverse effects.
[+] "Knowledge about TB" coded as Yes if any listed measure was answered "Yes" for the questions: Is TB Communicable, Transmissible through air (Coughing, sneezing, speaking, bacteria), through contaminated food/water, through surface, Other(s), Is TB Curable?
[§] "Knowledge of TB prevention" coded as Yes if any listed measure was answered "Yes" response to the following variables: Use of masks, keeping distance from other persons with active TB, maintaining hygiene, IPT.
